# The Use of Extended Reality Distraction Methods During Needle‐Related Procedures in Pediatric Hospital Care—Children's Experiences

**DOI:** 10.1111/jspn.70021

**Published:** 2026-07-29

**Authors:** Anna Stålberg, Johanna Granhagen Jungner, Cecilia Bartholdson, Henrik Hjelmgren

**Affiliations:** ^1^ Astrid Lindgren's Children's Hospital Karolinska University Hospital Stockholm Sweden; ^2^ Department of Women's and Children's Health Karolinska Institute Stockholm Sweden

**Keywords:** augmented reality, child‐centered care, distraction, mixed methods, needle‐related procedures, pediatrics, venipuncture, virtual reality

## Abstract

**Purpose:**

To describe children's experiences of using extended reality (XR) distraction, namely virtual reality (VR) and augmented reality (AR), during needle‐related procedures.

**Design and Methods:**

An exploratory, prospective convergent parallel mixed‐methods design was employed. Self‐reported measures and structured observations conducted in accordance with a predefined protocol were combined with semi‐structured interviews. Data were presented using descriptive statistics and synthesized through thematic analysis.

**Results:**

Observations were conducted during 29 venipunctures in children using XR distraction. A majority of the children (90%) reported positive experiences with the use of XR distraction. Numerical Rating Scale pain scores (0–10) were low in both groups, with a mean of 1.26 (SD 1.16) for VR and 1.77 (SD 1.79) for AR. Most procedures (62%) were carried out as planned. No child declined the use of the device. Physical restraint was not observed; however, supportive holding was used in 10% of procedures. Four themes were identified through the interviews: *Emotional support and comfort*, *The artificial world becomes reality*, *A diversified experience*, and *Usability aspects influencing user experience*.

**Conclusion:**

When appropriately implemented, XR distraction may reduce pain and anxiety among children during needle‐related procedures. However, it is not universally effective, underscoring the importance of a child‐centered approach that allows children to select their preferred distraction method. Further research is required to determine optimal implementation strategies, assess cost‐effectiveness, and clarify the role of XR distraction in delivering high‐quality pediatric care during needle‐related procedures.

**Practice Implications:**

The demonstrated benefits of XR distraction during needle‐related procedures may encourage healthcare professionals to incorporate such tools into clinical practice. Nevertheless, the findings also emphasize the importance of adopting a child‐centered approach, recognizing that XR distraction is not suitable for all children.

## What is currently known?

- Needle‐related procedures are widely recognized as a significant source of distress for children during hospitalization
- Virtual reality (VR) distraction has been shown to be an effective intervention for reducing distress in children undergoing needle‐related procedures.

## What does This article add?

- This study extends existing knowledge by examining augmented reality (AR) as a distraction intervention for needle‐related procedures and explores the experience from the child's perspective. The findings suggest that AR, like VR, can be an effective distraction for many children while highlighting the importance of considering individual preferences and experiences.

## 1 Introduction

Children visiting healthcare settings often undergo various painful medical procedures, with needle‐related procedures being the most common, including insertion of a peripheral intravenous catheter, venous blood sampling, or injections (Harnik and Moreiras 2014; Reigart et al. 2012). Henceforth, the term needle‐related procedures will be used to cover all such procedures.

Needle‐related procedures are essential to make correct diagnoses and provide relevant treatment for children with an acute or chronic disease. However, children describe these procedures as the most distressing events during their hospital stay (Kennedy et al. 2008; Walther‐Larsen et al. 2017). Nevertheless, needle‐related pain in children is often unrecognized and undertreated, which can lead to needle phobia, pre‐procedural anxiety, hyperalgesia, and avoidance of healthcare, resulting in increased morbidity and even mortality (Goubert and Friedrichsdorf 2020). Pain, fear, and anxiety are emotions present before, during, and after a needle‐related procedure and risk affecting children's well‐being and cause short‐ and long‐term consequences (Bahorski et al. 2015; Karlsson et al. 2016; McMurtry et al. 2015). Fear and pain are also closely linked, meaning that increased fear can trigger a pain sensation. However, the opposite situation can arise as well (McMurtry et al. 2015).

Non‐pharmacological methods during needle‐related procedures, used in addition to pharmacological ones, are crucial for providing optimal care and for reducing distress for the child (Birnie et al. 2014). Distraction is one such non‐pharmacological method (Erdogan and Aytekin Ozdemir 2021; Leroy et al. 2016; Sadeghi et al. 2013) and takes the form of cognitive interventions that help the child to shift focus from the painful procedure to a more rewarding activity. Distraction can either be active or passive, although the active version seems to reduce pain and anxiety more effectively (Shen et al. 2023). Extended reality (XR) is an umbrella term for different immersive technologies, including virtual reality (VR) and augmented reality (AR). The VR technology allows users to immerse themselves in a virtual world, passively or actively, without being able to see what is happening around them. AR technology allows users to see the real world and simultaneously interact with a digital animation. Although a relatively modern distraction method, VR has been evaluated for reducing distress in several medical contexts and for various procedures (Addab et al. 2022; Cáceres‐Matos et al. 2024; Goldsworthy et al. 2023; Thybo et al. 2022). AR represents a more recent technology. Hence, extensive research is still lacking, although relatively newly performed research indicates promising results (Caruso et al. 2021; Sekeler et al. 2025; Yoo and Son 2024).

This study, focusing on children's experiences, forms part of a larger project aiming to gain knowledge from the perspectives of children, parents, and healthcare professionals on experiences of XR distraction used during needle‐related procedures. As stated above, there is an already existing body of knowledge regarding the use of VR for distraction during these kinds of procedures. However, its effect on pain and fear can be either positive (Özalp Gerçeker et al. 2020) or negative (Thybo et al. 2022), leading to the clinical importance of knowing when VR distraction is useful or not. Furthermore, the evidence concerning AR distraction and children's own experiences of using the technology is evidently limited. Therefore, the aim of this specific study was to describe children's experiences of using XR distraction during needle‐related procedures.

## 2 Methods

### 2.1 Design

An exploratory, prospective convergent parallel mixed‐methods design was employed. The quantitative data were collected through self‐reported measures and structured observations. The qualitative part comprised semi‐structured interviews. The study is reported in accordance with the COREQ checklist (Tong et al. 2007).

### 2.2 Settings and Recruitment Process

The study was conducted in a pediatric emergency department (PED), as well as outpatient and inpatient units at a tertiary hospital in the capital region of Sweden. Purposive sampling was employed to obtain rich and informative data, enabling the inclusion of a heterogeneous group with respect to age and gender (Patton 2015). The initial plan was to recruit 10 children from each setting (a total of 30 participants), divided into two equally sized groups (15 + 15) using either VR or AR. The target of 10 children was achieved in two of the three settings, while nine children were recruited from the PED because of a logistical issue. For each procedure, children were allocated to either VR or AR distraction based on personal preference; no randomization was applied. Inclusion criteria comprised children aged 5–17 years undergoing a needle‐related procedure and able to understand and speak Swedish. Children with epilepsy, nausea, head injuries or severe trauma, ongoing hallucinations, or cognitive impairment were excluded, as these conditions might be incompatible with the XR experience.

### 2.3 XR Interventions

In this study, a PICO 2 headset was used for VR distraction. Interactive games requiring one‐handed control were primarily used; however, one participant selected a non‐interactive VR film due to limited arm mobility (orthopedic reason). The VR solution offered multiple games, and the one to use was chosen collectively between the research team and the child. For AR distraction, the Magic Leap 2 headset was utilized. A customized AR game was installed, designed to be accessible across all age groups included in the study. That game aimed to engage the child by watering plants that appeared in the room. When watering the plants they grew and, eventually, filled the room. All children were given the opportunity to familiarize themselves with the VR or AR device prior to the procedure, and the devices were typically applied a few minutes before the procedure commenced. The researchers introduced the devices and provided guidance on their use before, during, and after the procedure. Children were permitted to remove the device during the procedure if they wished and could continue using it afterwards if desired.

### 2.4 Data Collection

A study‐specific questionnaire, developed in collaboration with a survey specialist, was used to explore children's perceptions of using VR or AR technology. Self‐report scales were employed to collect data on pain and satisfaction. Children younger than 7 years used the Faces Pain Scale‐Revised (FPS‐R), which employs a series of facial expressions to indicate levels of pain (Hicks et al. 2001). Children aged 7 years and older used the Numeric Rating Scale (NRS), ranging from 0 (no pain) to 10 (worst possible pain) (Pagé et al. 2012). In addition, a study‐specific observation protocol (see Supporting File 1), developed with inspiration from a compliance protocol by Caruso et al. (2020), was completed during the procedure by a member of the research team (AS, HH, or IK) present at the time. This protocol captured aspects such as child interaction and participation, duration of the procedure, and the number of venipunctures required.

Semi‐structured interviews were conducted to explore children's experiences of using VR or AR as a distraction during needle‐related procedures (see Supporting File 2 for the interview guide). The interviews were conducted by two of the authors (AS and HH) and one research assistant (IK), audio‐recorded, and transcribed verbatim for analysis. Where necessary, probing questions were asked to elicit more detailed responses (Patton 2015).

The observations were conducted in the clinics described above. Needle‐related procedures (venipunctures) were performed as part of routine clinical care for diagnostic or treatment purposes. No procedures were performed specifically for the purposes of this study. Most interviews were conducted shortly after the procedure; occasionally, they took place later the same day. Interviews ranged from 4 to 12 min in duration, with a mean length of 7 min. In addition, background information, including age, sex, diagnosis, and prior experience of needle‐related procedures, was collected. Data collection took place between September and December 2023.

### 2.5 Data Analysis

**Figure 1:**
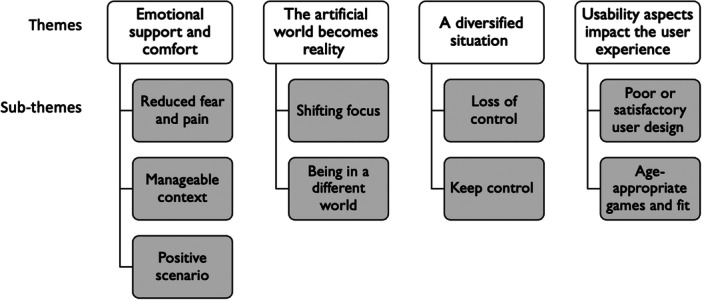
Thematic overview.

Quantitative data from the questionnaires and observation protocols were compiled in Microsoft Excel and analyzed descriptively using percentages, means, and standard deviations. Qualitative data were analyzed using an inductive reflexive thematic analysis approach, informed by Braun and Clarke (2006); Braun and Clarke (2020), to identify patterns in children's experiences of XR distraction. Initially, transcripts were read repeatedly to achieve familiarization with the data. As the study focused on XR distraction as a whole, meaning units were identified across all transcripts and compiled into a single dataset, irrespective of whether they referred to VR or AR experiences. Two members of the research team (AS and HH) independently coded a subset of interviews. To enhance trustworthiness and ensure consistency in the coding process, a reflexive discussion was held with a third researcher (JGJ). The remaining interviews were then divided among the three researchers and coded independently. Following completion of coding, the team met to identify and resolve discrepancies and reach consensus. Preliminary themes were then developed, followed by a review of both the codes within each theme and the relationships between themes across the entire dataset. This iterative process involved ongoing discussion within the research team and resulted in minor refinements, producing the final set of themes and subthemes (see Figure 1).

### 2.6 Reflexivity Statement

The research team comprised both early adopters and experienced users of XR technology. In addition, all members had expertise in child‐centered care, children's rights, and qualitative research methods.

### 2.7 Ethical Considerations

Ethical approval was obtained from the Swedish Ethical Review Authority (reference no. 2023‐03254‐01) prior to the commencement of the study. Before the needle‐related procedures and data collection, children and their parents received verbal information about the study from nursing staff in each setting. Following their agreement to participate, both children and parents were provided with written information by a member of the research team. They were also informed of their right to withdraw from the study at any time without any impact on future care. Written informed consent was obtained from the parents of children under 15 years of age. Children aged 15 years and older provided written consent independently. To ensure confidentiality, all interview data were coded and could only be linked to participants by the researchers involved in the study. No unauthorized individuals had access to the data.

## 3 Findings

In the findings section, both quantitative and qualitative findings are presented. The quantitative component reports data from the VR and AR groups separately to clarify children's perspectives on the specific technology used. The qualitative component provides an integrated account of children's experiences of XR (VR and AR) distraction during needle‐related procedures. At the outset, contextual information derived from the interviews is presented.

### 3.1 Contextual Findings

From the interviews, it was identified that most of the children reported feelings of nervousness regarding needle procedures. Additionally, some of them expressed stress and a need for preparation before the actual procedure commenced. These feelings arose either at home or upon approaching or entering the hospital. Irrespective of the use of topical anesthetic cream, children described experiencing fear prior to the procedure, either as fear of pain or fear itself. Children without prior experience reported uncertainty about what to expect as the primary source of fear, whereas those with previous experience often feared difficulties with venous access. However, not all experiences were negative. Familiarity with the procedure helped some children to normalize the experience, although it did not render it pleasant. A small number of children described the procedures as positive, perceiving intravenous treatment as less painful than alternative injection methods. Additionally, some children viewed the hospital visit positively as it provided a legitimate reason to be absent from school.

### 3.2 Quantitative Findings

In total, 29 children participated: 9 from the PED, and 10 each from the outpatient and inpatient units (see Table 1). The needle‐related procedures predominantly involved peripheral venous catheterization (*n* = 28), with one case involving venous blood sampling. Most children (*n* = 22) had prior experience of such procedures. Almost all children (90%) reported that XR distraction helped them feel less frightened during the procedure (VR 94%; AR 86%). Approximately two‐thirds of the children using VR reported feeling very well during the procedure, and nearly 80% of the children using AR reported similar experiences. The vast majority (93%) of children using VR, and approximately seven out of ten using AR, indicated that they would choose the same distraction method for a future procedure. Pain scores were low in both groups; however, the AR group demonstrated a wider range (0–6) compared with the VR group (0–3). Further details are presented in Table 2.

**Table 1:** Participant background data and frequency of VR and AR use.

Category	VR	AR	Total
Children *n* (%)	15 (52)	14 (48)	29 (100)
Age range (mean/SD)	5‐13 (10/2.55)	6‐17 (12/3.02)	5–17 (11/2.95)
Childhood 5–9 years *n* (%)	6 (40)	2 (14)	9 (28)
Adolescents 10–18 years *n* (%)	9 (60)	12 (86)	21 (72)
Gender			
Girl *n* (%)	10 (67)	6 (43)	16 (55)
Boy *n* (%)	5 (33)	8 (57)	14 (45)
Children in each setting			
Outpatient clinic *n* (%)	5 (33)	5 (36)	10 (34)
Pediatric emergency department *n* (%)	5 (33)	4 (28)	9 (32)
Inpatient ward *n* (%)	5 (33)	5 (36)	10 (34)
Reason for visit			
Iv treatment (rheumatic patients) *n* (%)	4 (27)	6 (43)	10 (34)
Infections (GE, UTI, unclear fever) *n* (%)	1 (7)	2 (14)	3 (10)
Orthopedic problems (surgery, reposition of fractures) *n* (%)	7 (47)	2 (14)	9 (31)
Other (various kinds of pain, diabetes, depression) *n* (%)	3 (20)	4 (29)	7 (24)
Earlier experiences of needle procedures			
Yes *n* (%)	10 (67)	12 (88)	22 (76%)
No *n* (%)	5 (33)	2 (12)	7 (24%)
Use of local anesthetic			
Yes/No	15/0	10/3	25/3
EMLA cream *n* (%)	12 (80%)	10 (100%)	
Rapydan *n* (%)	3 (20%)	0	
	1 child used both	1 missing data	
Disrupted procedure *n* (%)	1 (7)	2 (14)	3 (10)
Length of procedure in minutes (range)	2–30	2–15	2–30
Mean (SD)	8.7 (7.52)	6.2 (3.41)	
Number of venipunctures *n* (% of all children)	1 (80)	0 (7^1^)	
	2 (13)	1 (85)	
	4 (7)	2 (8)	
		1 missing data	

1 One child refused having the IV line inserted while using the AR headset. Instead, she preferred to use an iPad. However, she kept refusing the IV line although watching her iPad. Hence, no venipuncture was performed. Despite not following the original protocol, this child continued her study participation.

**Table 2:** Children satisfaction survey.

Question	VR *n* = 15	AR *n* = 14
How did you feel about wearing the VR/AR device? 1. Very uncomfortable 2. A little uncomfortable 3. Comfortable	Mean/SD: 2.86/0.53	Mean/SD: 2.78/0.43
	*n* = 1 (7%)	*n* = 0 (0%)
	*n* = 0 (0%)	*n* = 3 (21%)
	*n* = 14 (93%)	*n* = 11(79%)
Did you feel scared before the procedure? 1. Very scared 2. A little scared 3. Not scared at all	Mean/SD:2.53/0.64	Mea/SD: 2.28/0.61
	*n* = 1 (7%)	*n* = 1 (7%)
	*n* = 5 (33%)	*n* = 8 (57%)
	*n* = 9 (60%)	*n* = 5 (36%)
Did the glasses make you feel more or less scared during the procedure? 1. Much more scared 2. A little more scared 3. No difference 4. A little less scared 5. A lot less scared	Mean/SD: 4.60/0.63	Mean/SD: 4.21/0.89
	*n* = 0 (0%)	*n* = 0 (0%)
	*n* = 0 (0%)	*n* = 1 (7%)
	*n* = 1 (7%)	*n* = 1 (7%)
	*n* = 4 (27%)	*n* = 6 (43%)
	*n* = 10 (67%)	*n* = 6 (43%)
How did the experience of the procedure itself feel? 1. Felt not good at all 2. Felt little good 3. Felt very good	Mean/SD: 2.70/0.45	Mean/SD: 2.71/0.61
	*n* = 0 (0%)	*n* = 1 (7%)
	*n* = 4 (27%)	*n* = 2 (14%)
	*n* = 10 (67%)	*n* = 11 (79%)
	1 missing data	
How painful was the procedure itself?	Mean/SD: 1.26/1.16	Mean/SD: 1.77/1.79
0 = no pain	Min: 0	Min: 0
10 = worst pain ever	Max: 3	Max: 6
		1 missing data
Would you want to use the glasses again if you were to undergo a similar procedure? 1. No 2. Maybe 3. Yes	Mean/SD: 2.87/0.52	Mean/SD: 2.50/0.90
	*n* = 1 (7%)	*n* = 4 (31%)
	*n* = 0 (0%)	*n* = 0 (0%)
	*n* = 14 (93%)	*n* = 9 (69%)
		1 missing data

Observational data showed that most procedures (90%) were conducted as planned. In three procedures (10%), interruptions required the removal of the XR headset. Reasons included a participant requesting a break following multiple puncture attempts (*n* = 4), a preference for an alternative distraction method, and a technical malfunction of the headset. All children interacted well with the XR technology; none refused or attempted to avoid using the devices, and none cried when the devices were applied. No physical restraint was observed; however, supportive holding of the arm was used in three procedures (10%).

### 3.3 Qualitative Findings

The thematic analysis generated four themes: *Emotional support and comfort*, *The artificial world becomes reality*, *A diversified experience*, and *Usability aspects influencing user experience*, each with associated subthemes (see Figure 1).

### 3.4 Emotional Support and Comfort

Children described XR distraction as helping them manage distressing sensations and rendering the situation more controllable. This theme comprises the subthemes *Reduced fear and pain*, *Manageable context*, and *Positive scenario*.

#### 3.4.1 Reduced Fear and Pain

When using XR distraction, children perceived needle‐related procedures as less frightening and, consequently, less painful. Feelings of worry and distress were reduced or, in some cases, eliminated entirely, as the interactive and immersive VR or AR games engaged their attention through enjoyable and distracting activities.

Interviewer: How do you usually react to a needle‐related procedure?

Child: Most of the time I hug my mum and sometimes I scream, “Ouch!” Perhaps that makes it worse because then I move my arm. But this time I almost felt nothing.

(Girl, 10 years, AR, outpatient unit)

The use of VR devices enabled children to disengage from the hospital environment, which they otherwise commonly associated with needle‐related procedures. Although most children perceived XR distraction as effective in reducing pain, there was notable individual variation in how this reduction was experienced. Some children reported that pain disappeared entirely or was reduced to a minimal level. Others remained aware of the procedure but did not interpret the sensation as painful. A further group described only limited pain relief, indicating that they continued to experience both pain and fear during the procedure.

#### 3.4.2 Manageable Context

When using the VR device, the real‐life environment and the people present become less salient. Despite this, and largely due to the awareness that a parent was present in the room, albeit not visible at that moment, no children reported feelings of fear. Instead, there was a general acceptance of not being able to see their parents. While immersed in the VR games, most children noticed that something was happening to their arms. Although they recognized this sensation as the needle touching the skin, they accepted the situation and were able to remain calm, while still feeling adequately distracted. In contrast to VR, AR allowed children to see the actual room and, consequently, the procedure being performed. Most children appreciated being able to view the entire environment rather than only a screen, as this contributed to feelings of safety.

Child: It was good. I liked not just watching the screen but seeing the whole room and everything. I liked that.

(Boy, 9 years, AR, PED)

The additional digital elements presented through the AR glasses were perceived as helpful in directing attention towards the game, whilst simultaneously allowing children to observe the needle insertion.

#### 3.4.3 Positive Scenario

The use of VR or AR devices provided a satisfactory distracting effect and was therefore well received by the children, although their enthusiasm was often expressed briefly using words such as “good,” “positive,” or “cool.”

Interviewer: What was it like to use the VR device?

Child: Very good!

(Boy, 10 years, VR, PED)

There was a sense of joy and satisfaction in having the opportunity to try VR or AR, and XR distraction was perceived as a fun addition to the situation. This was particularly evident among children who did not have any alternative preferred distraction methods. This positive perception of XR distraction led some children to express a willingness to recommend this technology to others. However, children who were initially somewhat reserved or hesitant towards VR or AR often managed to overcome their initial skepticism. Overall, no negative aspects were identified by the children in relation to their experience of XR distraction. Children who had tested AR but also had prior experience of VR commented on the advantages and disadvantages of each approach without expressing a clear preference. Instead, they emphasized the importance of considering the individual child's needs and preferences in each situation to determine the most appropriate form of distraction.

### 3.5 The Artificial World Becomes Reality

Children's experiences of XR distraction facilitated a shift in attention away from the ongoing procedure. Instead, they became engaged in the games, experiencing immersion in an alternative world, as described in the subthemes *Shifting focus* and *Being in a different world*.

#### 3.5.1 Shifting Focus

Children described being distracted by either VR or AR. While they remained aware of the nurse performing the procedure, they did not focus on the needle insertion itself. As their attention was directed towards the VR or AR game, they reported little or no perception of pain or anxiety. Furthermore, when attention was not directed towards the procedure, children described feelings of enjoyment and amusement, which helped shift focus away from both the procedure and the anticipation of pain. XR distraction was described as supporting calmness and concentration, and as providing a sense of protection from a negative experience.

Interviewer: Tell me what you found positive about using the AR device.

Child: I found it so real. You could get distracted and forget what was happening around you. If something bad happens, you will not notice because you are in the middle of a game.

(Girl, 13 years, AR, inpatient unit)

#### 3.5.2 Being in a Different World

Children described how XR distraction enabled them to enter a game‐based world. This sense of being in a parallel environment helped them to disengage from reality and to shift attention away from the procedure, as well as the associated fear and pain. In addition, the digital environment was sometimes perceived as more homely, allowing children to momentarily forget that they were in a hospital setting.

Interviewer: In what way do you think the glasses affected you when you had your blood test?

Child: I felt like I was in a completely different world, like I was just sitting at home playing and not here. It was cool.

(Girl, 11 years, VR, outpatient unit)

In some cases, however, children reported feeling overwhelmed by the VR or AR content, which led to negative experiences. Certain games were perceived as overly demanding, resulting in reduced concentration on the virtual environment and increased focus on the real‐life procedure.

Interviewer: What do you think distracts you best?

Child: At the end there were too many flowers, so I could not concentrate on the game

and instead, I focused more on reality

(Girl, 15 years, AR, outpatient unit)

### 3.6 A Diversified Situation

Children's experiences of XR distraction varied depending on individual needs and preferences in the situation, as reflected in the subthemes *Loss of control* and *Keep* c*ontrol*.

#### 3.6.1 Loss of Control

When using VR, children became immersed in the interactive virtual environment presented within the headset. Some found this experience acceptable but unusual, particularly due to their inability to see the surrounding room. Other children, especially those who preferred a sense of control, expressed a preference for AR over VR.

Interviewer: What was it like not being able to see the room?

Child: It felt weird

(Boy, 11 years, VR, outpatient unit)

Not being able to see the room also meant being unable to observe the procedure. Awareness that something would happen, without knowing when or how, led to feelings of fear, stress, and tension in some children, although others still found the experience acceptable. In such situations, there was often a strong need for verbal preparation, for example, through counting prior to the procedure.

Interviewer: Would it have been better if they said, “Now I will start” or count “1, 2, 3”?

Child: Yes, I would prefer them to tell me when they begin, so I know.

(Boy, 10 years, VR, PED)

#### 3.6.2 Keep Control

When using AR, children were able to orient themselves within the room and, if they wish, follow the procedure. This ability to remain aware of events contributed to a sense of control over the situation, which was particularly important for some children. These children preferred to remain aware of what was happening rather than using a distraction method that prevented them from following the procedure, which they felt could otherwise result in sudden and unprepared pain responses.

Interviewer: You felt that you wanted to see what happened?

Child: Yes. It was positive because I could watch the needle when it was inserted into the blood vessel.

(Boy, 10 years, AR, inpatient unit)

Both coping ability and perceived control were enhanced when AR was used. Children reported remaining calm despite observing and being aware of the procedure. Similarly, the AR game contributed to a sense of calmness, as it was perceived as enjoyable.

### 3.7 Usability Aspects Impacting the User Experience

Usability factors influenced children's experiences of XR distraction, as described in the subthemes *Poor or satisfactory user design* and *Age‐appropriate games and fit*.

#### 3.7.1 Poor or Satisfactory User Design

Some children reported that VR games functioned poorly and were difficult to exit while lying down, particularly as head movements were required to do so. Although designed to provide distraction during needle‐related procedures, some games were considered too short and therefore not well aligned with the duration of the procedure. In addition, some graphics were difficult to interpret.

Interviewer: What did you think was bad about using them?

Child: I think they were a bit… sometimes it was difficult to see things, and it was a bit unclear, but otherwise they were good!

(Girl, 17 years, AR, inpatient unit)

Some nausea and dizziness occurred, which made the XR experience less pleasant. Nevertheless, they still generally describe VR or AR as enjoyable. Moreover, there were differing views regarding the number of available games. Some children preferred a wider selection to allow more personalized distraction, whereas others considered the existing range sufficient. Children also appreciated that some games were adapted for those unable to use hand controls.

#### 3.7.2 Age‐Appropriate Games and Fit

As the study included children aged 5–17 years, with approximately two‐thirds being adolescents, a common view was that some games were more suitable for younger children. Despite this, children of all ages reported that the games provided an effective distraction during procedures. Some children reported poor device fit, describing the headset as too tight or too small, which resulted in discomfort and a desire to remove it. In some cases, an unstable fit allowed the real environment to become partially visible, which disrupted immersion and reduced the effectiveness of distraction.

Interviewer: How did it feel to wear the AR device?

Child: I think it was a bit too small.

Interviewer: In what way? Over your nose, on the top of your head, or at your neck?

Child: Everywhere

(Girl, 6.5 years, AR, inpatient unit)

## 4 Discussion

This study aimed to describe children's experiences of using XR distraction during needle‐related procedures. The novelty of this study is the adding of AR technology used for distraction. The findings indicated that children perceived XR distraction as a fun form of distraction and, overall, expressed positive attitudes towards its use. The application of VR or AR appeared to enhance the perceived controllability of procedures and to reduce both pain and fear in most children, although not in all cases.

A large majority of participating children had previous experience of needle‐related procedures. Nevertheless, they reported feelings of anxiety and distress in anticipation of the procedure. These findings are consistent with previous research indicating that needle‐related procedures are among the most distressing experiences for hospitalized children (Kennedy et al. 2008; Walther‐Larsen et al. 2019). To minimize the adverse consequences associated with such procedures, both in the present and in future care contexts, it is essential to address emotions such as fear, pain, and anxiety (Bahorski et al. 2015; Karlsson et al. 2016; McMurtry et al. 2015). Distraction is one strategy to mitigate these negative emotions; however, it may take many forms. The present study focused on XR distraction, encompassing both VR and AR. In several respects, the findings corroborate previous research demonstrating predominantly positive experiences, satisfaction, and reductions in fear and pain associated with VR distraction during needle‐related procedures (Caruso et al. 2020; Özalp Gerçeker et al. 2020; Thybo et al. 2022). A key contribution to this study is the inclusion of AR as a distraction modality. Unlike VR, which fully immerses the user in a virtual environment, AR enables simultaneous perception of the real world alongside engagement in a virtual activity. Children using AR reported that being able to see the room and the procedure contributed to feelings of safety and calmness. At the same time, the virtual game was considered essential in helping them to redirect attention when the real‐world situation became too distressing.

It is not surprising that some children who preferred to maintain control of the situation responded less favorably to VR. The inability to observe the environment elicited feelings of fear and stress, suggesting that a method intended to reduce distress may, for some individuals, have the opposite effect. These children, therefore, indicated that AR may be more appropriate, as it allows both distraction and situational awareness. These findings reinforce the importance of offering children a choice. Kleye et al. (2021) similarly highlight that children's perspectives may differ from those of adults, including parents and healthcare professionals, regarding the selection of distraction methods (Sommer et al. 2010). However, when supported by a responsive and reassuring adult environment, children are better able to select strategies that suit their individual needs and the clinical situation. Accordingly, and in line with Leroy et al. (2016), it is essential that healthcare professionals routinely develop individualized plans for preparation, pain management, and anxiety reduction. Such plans should incorporate the child's perspective, including preferences regarding XR distraction, to ensure that care is optimized in accordance with the child's best interests. By actively eliciting and respecting children's preferences, healthcare professionals adopt a child‐centered approach (Coyne et al. 2016), aligned with broader children's rights principles that uphold autonomy and dignity (Unicef 2024). Children's rights in relation to medical procedures have also been emphasized in the development of the iSupport standard, which highlights the importance of providing adequate support and ensuring child participation in procedural decision‐making to safeguard both immediate and long‐term best interests (Bray et al. 2023).

Although experiences were predominantly positive, some children reported usability‐related challenges that negatively affected their experience. Similar findings have been reported in previous studies, including issues such as unclear visual interfaces and difficulties with hand controls (Flegel and Mentler 2023; Nilsson et al. 2009), as well as age‐inappropriate XR content (Chan et al. 2019). Cybersickness, including dizziness and nausea, is another potential adverse effect of XR use, although it is rarely reported in pediatric VR studies (Chan et al. 2019; Thybo et al. 2022; Walther‐Larsen et al. 2019). For this reason, indicators of cybersickness were not included in the observation protocol used in this study. Nevertheless, when interviewed, one child reported mild dizziness, which did not necessitate discontinuation of the intervention or modification of the distraction method.

Although not identified as a major issue in this study, usability factors are important as they influence the overall user experience, and a negative experience may reduce the effectiveness of an otherwise beneficial intervention. The degree of immersion varies between VR applications, and differences in engagement may influence outcomes related to pain and fear (Tas et al. 2022). Therefore, careful consideration is required prior to implementing XR interventions in pediatric healthcare settings. While the findings indicate generally positive attitudes among children, it is important that healthcare professionals do not become uncritically enthusiastic about this technology. A balance between technological and human interaction must be maintained, and core values such as the child's growing autonomy and dignity should be preserved within a child‐centered design approach (Bruno et al. 2022). Children represent a vulnerable group, and the therapeutic relationship should not be replaced by technological solutions, as inappropriate use may be counterproductive. Healthcare professionals should therefore critically reflect on the purpose of implementing such interventions. From a clinical perspective, however, the introduction of a well‐received distraction method may shift the focus from procedure‐centered to child‐centered care (Stålberg et al. 2026), potentially reducing the challenges faced by pediatric nurses in managing needle‐related procedures, including procedural accuracy and first‐attempt success rates (Hjelmgren et al. 2022; Kleidon et al. 2019; Piazza et al. 2019).

Although VR and AR were used as distinct interventions, the study was not designed to compare them directly. Instead, this study sought to establish a unified understanding of the VR and AR technologies utilized for distraction. However, the inclusion of AR has contributed to an enhanced understanding of how children perceive the use of this particular technology when undergoing needle‐related procedures. Nevertheless, self‐reported data indicated a slight advantage for VR. VR was also perceived as marginally more comfortable and more effective in reducing fear and pain, which is somewhat unexpected given that AR headsets were lighter and ergonomically designed. These findings were not adjusted for age or XR content, which may partly explain the observed differences. Despite this slight advantage for VR, both modalities were associated with high levels of satisfaction, suggesting that AR represents a promising option for future pediatric applications.

### 4.1 Limitations

The study design, which included a substantial qualitative component, enabled a comprehensive exploration of children's experiences. Participants were recruited from three clinical units, allowing a diversity of perspectives. Many findings align with previous research, suggesting potential transferability to other settings and populations. However, the study was conducted in a single tertiary hospital in Sweden, which may limit generalizability. All interviews were conducted by members of the research team who also introduced the XR devices and were present during procedures. This may have influenced children's willingness to express negative views. In addition, some interviews were conducted by a researcher with limited experience, although experienced in communicating with children. The study‐specific questionnaire included items with varying levels of response nuance. A more consistent response format across all items might have enabled a more detailed understanding of children's perceptions. Furthermore, the level of immersion and interaction differed between XR applications, which may limit comparability with other studies. Quantitative data were utilized to describe children's views on the use of VR or AR as a form of distraction (see Table 1). These data were not intended for use in group comparisons; consequently, no statistical testing was undertaken. In addition, the qualitative analysis was conducted using all available data, irrespective of whether it related to VR or AR. However, Table 1 indicates differences between the two groups that might have influenced the results, had the aim been to compare children's perspectives on VR versus AR distraction.

### 4.2 Implications for Practice

Children's experiences of XR distraction were generally positive, suggesting that such technologies may be a valuable addition to existing distraction strategies in pediatric care. However, XR distraction was not suitable for all children, as some preferred alternative strategies or no distraction at all to maintain a sense of control. This underscores the importance of a child‐centered approach that supports individual choice.

Implementation of XR requires investment in devices and age‐appropriate software. AR systems currently entail higher costs than VR; however, evidence suggests that VR may be cost‐effective in healthcare settings (Delshad et al. 2018), which may also apply to AR. Potential benefits include reduced needle‐related fear and trauma, as well as improved procedural experiences, with possible broader societal cost savings, although these are difficult to quantify.

Further research is needed to determine optimal implementation strategies, evaluate cost‐effectiveness, and explore the role of XR distraction in a wider range of pediatric procedures. Future studies could also investigate the use of XR in combination with ultrasound‐guided peripheral venous catheterization in children with difficult intravenous access.

## 5 Conclusion

A range of distraction methods, both active and passive, can be used to support children undergoing needle‐related procedures. The findings of this study suggest that XR distraction is generally perceived as engaging, useful, and beneficial by many children, although not by all. Some children preferred full immersion in VR, which excluded awareness of the clinical environment, whereas others preferred AR, which enabled simultaneous awareness of surroundings and engagement in distraction. A further group preferred alternative strategies or no distraction. These findings highlight the importance of recognizing and respecting individual preferences. XR distraction should therefore not be considered a universal solution, but rather a valuable option within the repertoire of distraction techniques available in pediatric care.

## Author Contributions

A.S. and H.H. led the conceptualization of the study, the data collection, and curation. A.S. and H.H. performed the data analysis with input from J.G.J. A.S. and H.H. wrote the first draft of the manuscript with input from all co‐authors. J.G.J. and C.B. were involved in review and editing and approved the final version for publication. All authors have materially participated in the research and manuscript preparation.

## Conflicts of Interest

The authors declare no conflicts of interest.

## Supporting information


Supporting File 1

Supporting File 2

## Data Availability

The data that support the findings of this study are available on request from the corresponding author. The data are not publicly available due to privacy or ethical restrictions. The data that support the results of this study are available from the corresponding author upon reasonable request.
