# Supplementary material for: The Use of Extended Reality Distraction Methods During Needle‐Related Procedures in Pediatric Hospital Care—Children's Experiences
Source: J Spec Pediatr Nurs. 2026 Jul 29;31(4):e70021. doi: 10.1111/jspn.70021 (PMC13417549; doi:10.1111/jspn.70021)
Supplement: Supplementary file 1 — Supporting File 1 [file JSPN-31-e70021-s002.docx]

**Observation protocol: Procedure**

| Identification number | Date | |
| --- | --- | --- |
| Reason for attending hospital Priority Level (applicable only to children attending the Paediatric Emergency Department) |  | |
| Sex |  | |
| Age |  | |
| Presence of any underlying medical condition?  If yes, please specify | Yes | No |
|  |  | |
| Previous experience of attending the paediatric emergency department, outpatient, or inpatient units | Yes | No |
| Previous experiences of needle-related procedures  If yes, please indicate whether the experience was positive or negative | Yes | No |
|  |  | |
| Number of caregivers present |  | |
| Number of accompanying siblings |  | |
| Number of healthcare professionals present (excluding research team members) |  | |
| Type of needle-related procedure | Blood sample | Iv-line |
| Choice of distraction method | VR | AR |
| Was the procedure interrupted?  If yes, please state the reason | Yes | No |
|  |  | |
| Removal of goggles  If yeas, please state the reason | Yes | No |
|  |  | |
| Use of local anesthetic cream(Emla/Rapydan)  If yes, please specify the method and duration of application | Yes | No |
|  |  | |
| Use of pharmacological pre-medication  If yes, please specify the type | Yes | No |
|  |  | |
| Total duration of the procedure |  | |
| Total duration for which the child wore the VR/AR goggles |  | |
| Number of attempts |  | |

**Observations protocol: Compliance**

Please select the statement that best reflects the child’s condition and the course of the procedure. Circle your answers.

**Crying**

1. No crying
2. Sobbing
3. Crying with tears
4. Hysterical crying

**Turning the head away from the VR/AR goggles**

1. No turning away of the head
2. Partial turning away of the head, but still accepting use of the VR/AR goggles
3. Turning the head away
4. Refusal to use the VR/AR goggles

**Passivity** (in relation to interaction with the technology)

1. Completely passive
2. Somewhat passive
3. Not passive

**The procedure**

1. Proceeded according to plan
2. Proceeded almost according to plan
3. The procedure was interrupted

**Restraint**

1. No restraint
2. Minimal restraint
3. Moderate restraint
4. Full restraint

Please describe the type of restraint used (if applicable)

(The compliance protocol is an adaptation of the protocol described in *Virtual reality during pediatric vascular access: A pragmatic, prospective randomized, controlled trial* by Caruso et al., 2020).
