# Supplementary material for: The Use of Extended Reality Distraction Methods During Needle‐Related Procedures in Pediatric Hospital Care—Children's Experiences
Source: J Spec Pediatr Nurs. 2026 Jul 29;31(4):e70021. doi: 10.1111/jspn.70021 (PMC13417549; doi:10.1111/jspn.70021)
Supplement: Supplementary file 2 — Supporting File 2 [file JSPN-31-e70021-s001.docx]

**Interview Guide for Children**

**Introduction**
 At the outset, the interviewer should:

- introduce themselves
- explain how the interview will be conducted
- indicate the approximate duration of the interview
- inform the child that they may stop the interview at any time should they no longer wish to continue
- explain that the interview will be recorded

**Rapport-Building Phase**

1. How old are you?
2. What is the reason for your visit to the hospital today?
3. Have you previously had a blood test taken, or have you had an intravenous (IV) line inserted?

**Interview**

1. What were your initial thoughts when the doctor or nurse informed you about the blood test or IV line?
2. Can you describe what happened when the nurse took the blood sample or inserted the IV line?
3. How did you perceive that situation?
4. How did you find the needle procedure?
5. Can you describe your experience of using the VR/AR goggles?
6. Earlier, you mentioned feeling scared/worried/sad (use the child’s own words) when you were told about the blood test or IV line. In what way did the VR/AR goggles affect your feelings in that situation?
7. Can you describe what it was like not being able to see your mother or father, or what was happening in the room? *(For children who used VR goggles)*
8. What aspects of using the VR/AR goggles did you find positive?
9. Were there any aspects you did not like when using the VR/AR goggles?
10. What are your thoughts about the game you watched and interacted with in the goggles?
